# Children’s and adults’ use of fictional discourse and semantic knowledge for prediction in language processing

**DOI:** 10.1371/journal.pone.0267297

**Published:** 2022-04-28

**Authors:** Ruth Lee, Craig G. Chambers, Falk Huettig, Patricia A. Ganea

**Affiliations:** 1 Ontario Institute for Studies in Education, University of Toronto St. George, Toronto, Ontario, Canada; 2 Department of Psychology, University of Toronto Mississauga, Mississauga, Ontario, Canada; 3 Psychology of Language Department, Max Planck Institute for Psycholinguistics, Nijmegen, Netherlands; 4 Centre for Language Studies, Radboud University, Nijmegen, Netherlands; The Chinese University of Hong Kong, UNITED KINGDOM

## Abstract

Using real-time eye-movement measures, we asked how a fantastical discourse context competes with stored representations of real-world events to influence the moment-by-moment interpretation of a story by 7-year-old children and adults. Seven-year-olds were less effective at bypassing stored real-world knowledge during real-time interpretation than adults. Our results suggest that children privilege stored semantic knowledge over situation-specific information presented in a fictional story context. We suggest that 7-year-olds’ canonical semantic and conceptual relations are sufficiently strongly rooted in statistical patterns in language that have consolidated over time that they overwhelm new and unexpected information even when the latter is fantastical and highly salient.

## Introduction

Grade school children are veteran consumers of fantastical fiction. Books, movies, and other media present children with events that contradict their direct experience of the world, in ways both impossible (a talking sea sponge who lives in a pineapple) and highly improbable (Winnie the Pooh gets stuck in Rabbit’s narrow burrow, having eaten a lot of honey). However, we do not know how children’s knowledge of the world competes with fantastical events in real time. How robustly can children interpret impossible and improbable entities and events as a story unfolds, and what does this tell us about the ways in which children mentally represent information in the course of narrative comprehension?

During language interpretation, message-level meaning is constructed through the use of lexical, syntactic, and semantic cues [1]. One core requirement of linguistic processing is the task of identifying relevant thematic relations between the entities and events evoked in a sentence. Studies of visually-situated language processing have shown that comprehenders use such relations to predict upcoming linguistic input, and in turn direct their attention to compatible referents in the visual world (e.g., [2, 3]). For example, when hearing the sentence ‘The boy eats the big cake’ while looking at a scene containing a cake and a bird, adults and children as young as 2 years of age look to the cake while ‘eats’ is unfolding [4].

Children as young as 3 years of age can also use their prior knowledge of the relations between actions and agents to generate more sophisticated predictions, such as anticipating ’bone’ upon hearing "The dog hides" [5]. By the age of 5, children are also able to use a novel agent-event mapping to predict that it will recur during a subsequent event [6].

In adults, predictive language comprehension is also influenced by higher-order aspects of meaning involving physical, functional and situational relations between entities and events. These include behavioural goals [7]; linguistic and pragmatic judgements about why and how information is provided [8–10], and the capacities of agents and instruments [3]. Situation-specific information that is not part of the here-and-now at the moment of utterance, such as the actions an object has recently undergone, is also used [11].

Studies have also shown that adults can effectively bypass lexical and semantic relations based on real-world knowledge. Even when the discourse is fantastical, event related potentials (ERPs) show that adults do not encounter processing difficulties if the fictional world is a familiar one. When Tom the cat (of Tom and Jerry fame) is described as picking up a chainsaw, adults’ prior knowledge of Tom’s ability to engage in un-catlike actions entails only a mild N400 effect, reflecting the comparative ease of interpreting the description [12]. Indeed, once the features of a fictional world are established, *conventional* actions can be more difficult for adults to assimilate. Foy and Gerrig [13] asked adults to read stories about an ordinary person (e.g., a Boston police officer) or a familiar fantastical character (e.g., Superman). When reading about Superman, participants were slower to read text describing realistic events that were consistent with their real-world knowledge but were inconsistent with their story-world knowledge (e.g., dying from being shot) compared to when they read about events that were consistent with their story-world knowledge, but not their real-world knowledge (e.g., bullets bouncing off the character’s chest). For unfamiliar fantastical worlds, reading studies have shown that, although adults experience initial online processing difficulty on their first encounter with information from this world, the difficulty is rapidly overcome [14, 15]. Even when the fictional world is especially fantastical, a brief introduction to the knowledge-violating ‘facts’ of a novel story world (e.g., a talking yacht) is sufficient for adults to use that knowledge to inform online language processing [16].

In some circumstances, contextual information can override linguistic knowledge during young children’s real-time language processing. Several studies have demonstrated that for preschoolers, discourse prominence and topicality can override grammaticality during real-time language processing [17, 18] although other research has shown that, when interpreting sentences that contain syntactic ambiguities, preschoolers tend to privilege lexical information over situation-specific knowledge about the potential referents available in the visual world [19, 20]. Little is known, however, about the real-time processes and underlying mechanisms involved in children’s interpretation of fantastical discourse. Yazbec et al. [21] presented adults and children aged 5–10 with brief fictional stories in which fifteen human or animal agents such as a girl, a bird, and a pilot (including one fantastical entity, a witch) engaged in unexpected actions on patients. Approximately half of these actions were thematically plausible, although the semantic relations between verb and patient were less strong than would be an alternative (for instance, a pilot flying a kite; an astronaut wearing a t-shirt). The remainder were thematically implausible (for instance, a bird eating honey; a penguin catching a mouse). Following each story, listeners heard (e.g.) ‘The astronaut wears the t-shirt’, while looking at pictures that included both a t-shirt and a relatively more plausible referent, a spacesuit. Adults and 10-year-olds were more likely than were younger children to look proportionately more to the target (e.g., t-shirt) than to the global knowledge distractor (e.g., spacesuit), and log proportions of younger children’s looking time to the target relative to the global knowledge distractor suggested a distractor preference. Thus, children aged younger than 10 were not effective at privileging unexpected semantic information over their real-world knowledge during real-time sentence interpretation.

There are two possible explanations for the difference found by Yazbec et al [21] between the younger and older children. Young children may encounter a difficulty in strongly instantiating the unexpected information, depending more heavily than do adults on standard semantic associations that are expressed in the language they encounter as well as in the everyday events they experience in the real world. If so, it is possible that more salient contrasts between the everyday real world and a novel and engaging fictional world—such as one involving fantastical characters and thematically implausible information—could strengthen children’s representation of that information. This could, in turn, support children’s ability to integrate this information in real time as language is encountered. In this vein, recall advantages have been found for other kinds of story-disruptive material in both adults [22, interruptive actions; 23, sensational content] and children [24, 25]. Young children also find incongruity highly salient: watching an actor violate typical action-object relations is interesting and amusing to children as young as 3 (e.g., a person eating a houseplant [26]). Beyond preschool age, children also explicitly distinguish the boundaries between fantasy and reality. They judge impossible abilities to be associated with fantastical but not realistic characters [27], know to "quarantine" problem solutions presented in fantasy stories, and transfer solutions presented in realistic stories to the real world [28, 29]. Furthermore, when objects and events are ‘foregrounded’ in a narrative, this creates an expectation in adults that they will turn out to be relevant later [30], although it is not yet clear whether this is also the case for children.

A second possibility is that the unexpected information may be firmly instantiated in young children’s working memory, but they may lack the processing capacity to identify it as relevant in-the-moment, and so fail to manage the competing interpretations of a sentence as it unfolds [19, 31].

In order to adjudicate between these possibilities, the present study uses information that is not just unexpected, but overtly fantastical. More specifically, we use a spoken language eye tracking methodology to investigate children’s real-time language processing in discourse contexts that present not only thematically implausible events, but also fantastical protagonists. The goal of the study is to compare children’s and adults’ use of fictional information (which, in our paradigm, contradicts lexical and world knowledge) to guide predictions about upcoming language input. Can young children draw upon fantastical facts introduced in the prior discourse to predict upcoming referents? Further, how effectively does information drawn from a fantastical narrative compete with stored representations of actions and events in the real world?

We explore these questions by examining gaze patterns as children hear a discourse-final sentence in which the protagonist acts on an object in an unusual way (e.g., eating snow). If children draw to some extent upon the fantastical discourse to interpret the sentence, their gaze patterns upon hearing the verb (e.g., "eating") should reflect greater anticipation of visually-displayed objects that are congruent with the discourse (compared to a situation where no fantastical context was provided), and comparatively less consideration of other objects that are more strongly tied to the verb in question, as measured by the probability of fixations to the objects in question.

We test this hypothesis in 7-year-old children because they are highly experienced with narratives, yet appear to show difficulty integrating novel semantic information during sentence interpretation [21]. Indeed, by the end of their preschool years, children are sensitive to the causal structure of discourse [32, 33], can adopt aspects of a story character’s mental and spatial perspective [34–36], and begin to make inferences connecting the events evoked in narrative with world knowledge [37]. Together with grade school children’s competence in distinguishing fantasy from reality (as discussed above), this work suggests that by the age of 6 or 7, children could plausibly be able to use fantastical facts introduced in a narrative to guide the early moments of processing. By contrast, preschool (5-year-old) children are less skilled than are 7-year-olds in their recall of main narrative elements, identification of critical explicit information, and ability to connect information across discourse [38] and are more often willing to attribute unconventional behaviour to humans [27].

## Method

### Participants

Sixty-three 7-year-old children (*M*age = 7.4 years, *SD* = .31, range: 7.01–7.98; 31 girls) and 66 adults (*M*age = 24.99, *SD* = 5.4, range: 18.44–35.99; 35 women) participated. Inclusionary criteria were normal or corrected-to-normal vision, no history of diagnosis or treatment of cognitive, speech, language, hearing, or attentional issues, and hearing English spoken at home more than 75% of the time (children) or hearing English spoken at least 50% of the time in the home environment on a continuous basis since birth (adults). Data from 33 additional participants were collected, but not used due to: language background (3 adults, 3 children), unsuccessful calibration (3 adults, 1 child), cases where no experimental trials captured eye movements during the verb windows of the critical sentences more than 50% of the time (7 adults, 5 children), failed pre-test (3 children), lack of attention (2 adults, 3 children), and misunderstanding the task (1 adult, and 2 children).

Data collection took place at a large public university in North America. Children were recruited from a family volunteer participant pool and, for the norming study, from a large local Science Centre. Adults were recruited from on-campus advertising. As a thank you, children received a small toy and adults received either a $5 CAD gift card or course credit. Most child and adult participants came from White and Asian middle-class backgrounds.

Ethical approval was received from the Social Sciences, Humanities, and Education Research Ethics Board at the University of Toronto, protocol reference 27966. Written consent was obtained from adult participants and from parents or guardians of child participants. Verbal consent was obtained from child participants. The study conformed to the standards of the Declaration of Helsinki: US Federal Policy for the Protection of Human Subjects.

Power analyses were conducted with alpha and power at the conventional levels of .05 and .8 respectively, and presumed large effects of discourse-congruence on sentence processing (based on the findings of Filik [12], Foy & Gerrig [13], and Nieuwland & Van Berkum [16]). Power analyses yielded a required sample size of N = 26 for each age group in each condition. Thirty-one 7-year-olds and 33 adults participated in the experimental condition, which presented listeners with a brief discourse describing protagonist actions (five sentences) prior to the target sentence. A separate group of 32 7-year-olds and 33 adults participated in a control condition in which target sentences were presented with only one sentence of discourse giving the name and identity of the protagonist (see below). Participants in both the experimental and control conditions heard two types of trial: the core test cases in which the target sentence included a semantically constraining verb (‘constraining verb trials’), and those in which the target sentence included a verb that was not semantically constraining (‘neutral verb trials’). Thus, the nature of the discourse context was a between-participants manipulation, and verb type was a within-participants manipulation for the experimental group.

### Materials

**Fig 1:**
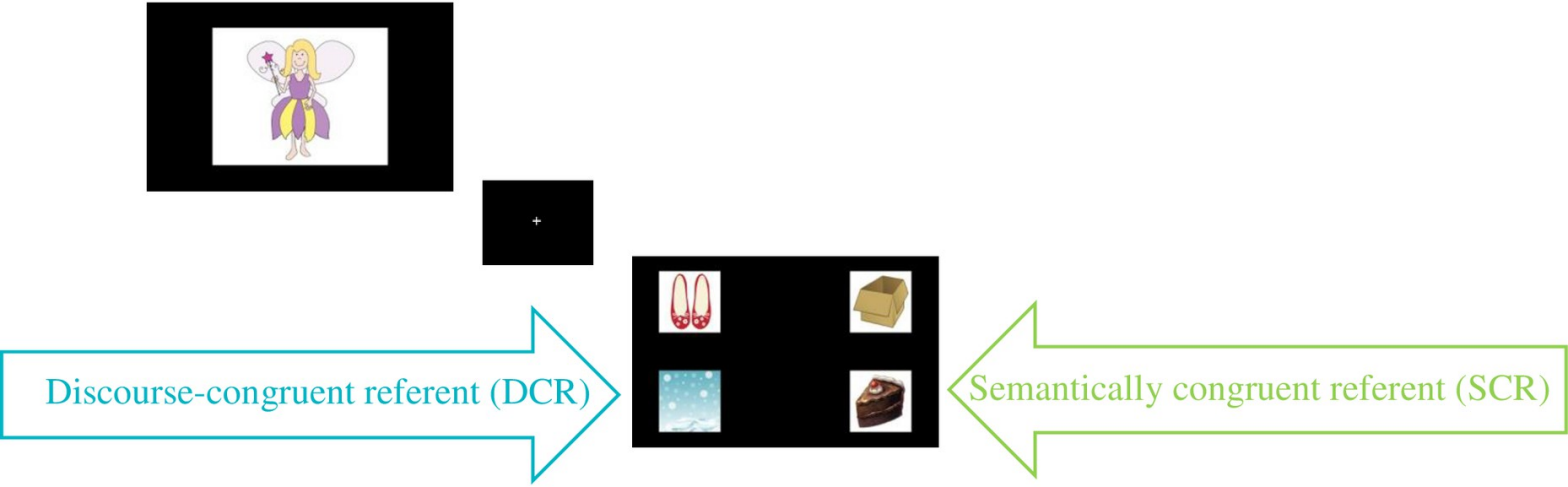
Example of stimuli used for the sentence comprehension task.

Stimuli comprised brief pre-recorded stories narrated by a female, native Canadian-English speaker in a child-directed voice, followed by a critical sentence. Displays accompanying each story depicted agents and objects within individual white boxes that were presented against a black background. Pictures were cartoon images taken from open-source resources. In the experimental condition, stimuli comprised 8 trials (described in S1 Appendix) which presented brief stories followed by a critical sentence. In the control condition, stimuli comprised 8 trials in which the critical sentence was preceded only by the introduction of the story character. In both conditions, there were also 8 additional filler trials. Each trial began with a description phase, which either presented a brief story about the pictured fantastical character (experimental condition) or merely introduced the character by name (control condition). This was followed by the test phase, in which listeners heard about a specific action performed by the character. In the description phase of the experimental condition, participants saw a centrally presented picture of a fantastical agent (e.g., a fairy, superhero or monster: see Fig 1) while hearing a story that contrasted objects that were semantically congruent (expected) with a given verb with referents that were unusual for that verb (e.g., ‘Chloe the fairy doesn’t have cake for her snack. She has snow for her snack! And Chloe doesn’t wear shoes on her feet. She wears boxes on her feet! What is Chloe going to do?’). Participants in the control condition saw the picture of the agent, but in place of the story they only heard (e.g.) ‘This is Chloe the fairy’. For both conditions, the image of the agent was then replaced with a central fixation cross. In the subsequent test phase, participants saw a display comprising the four mentioned items (e.g. cake, snow, shoes, and box), one placed in each corner of the screen (Fig 1). The critical sentence was then played, with the onset of the verb timed at 3500 ms after the appearance of the test phase display. This resulted in a period of approximately 2500 ms before the critical sentence began, during which participants explored the visual display with no verbal input. In both the experimental and control conditions, in 4 of the 8 trials, the verb in the critical sentence was semantically constraining (henceforth, ‘constraining verb trials’: e.g., ‘Chloe is eating up the snow.’) Thus, the verb could allow attention to be narrowed to an object in the display that was congruent with children’s stored semantic knowledge (henceforth, semantically congruent referent, or SCR: e.g., cake) and to an object that was congruent with the fantastical story information (discourse-congruent referent, or DCR: e.g., snow). In the other 4 trials, the verb did not constrain the referent (henceforth, ‘neutral verb trials’: e.g. ‘Chloe is looking at the snow.’) The verbs used in the story (e.g., ‘Chloe the fairy doesn’t *have* cake…’ were never repeated in the critical sentence (e.g., ‘Chloe is *eating up*. . .’). In the 8 filler trials, participants heard that agents ‘sometimes’ performed expected actions and ‘sometimes’ performed actions that violated world knowledge, breaking up the pattern in the content and outcomes of the stories and reducing the risk of strategic adjustments.

### Norming of stimuli

Offline tasks with a separate group of 4- and 5-year-old participants were conducted to establish that even younger children could recognize the objects and used the verbs to identify referents in the expected manner. Children were tested at a large local Science Centre. The experimenter showed the child a four-object display, provided a label, and asked her to identify the relevant object. 100% of children recognized all the images presented during the critical sentences (N = 8 per target image). Children were then introduced to pictures of agents (e.g. ‘This is Chloe the fairy’), following which they were presented with a four-object display, and asked (e.g.) ‘What will Chloe eat?’ Four- and 5-year-olds selected the only referent that was semantically plausible following the verb over 90% of the time across trials (N = 16 per target image). Example images for a given trial are shown in Fig 1. The object that was coded as the semantically plausible one for the norming task is the one labelled as the semantically congruent referent.

We used latent semantic analysis (LSA) [39] to confirm the strength of the underlying semantic relation between the verbs used in the stories and each of their potential noun complements (e.g., the objects labelled as the semantically congruent referent and the discourse congruent referent in Fig 1). The pairwise, term-to-term comparison method was selected on the Latent Semantic Analysis website (lsa.colorado.edu) to retrieve cosine measures for every typical verb-noun pairing (e.g., ‘eating’ and ‘cake’) and every unusual verb-noun pairing (e.g., ’eating’ and ’snow’) to be used in the eventual narratives. Cosine measures have a minimum of -1 and a maximum of 1. Paired samples t-tests for cosine measures using the ’General Knowledge up to Third Grade corpus’ (appropriate for 7-year-olds) confirmed that relatedness measures for typical pairings (*M* = .23, *SD* = .14) were significantly larger than those for unusual pairings (*M* = -.7, *SD* = .06, *t*(15) = 3.82, *p* = .002).

### Procedure

#### Offline pre-trials

To confirm that children could recall details from a simple five-sentence discourse of the type used in the experiment, the main task was preceded by two offline pre-trials in which children were asked a comprehension question. For instance, children heard ‘Gordon the gnome doesn’t bang on a drum. He bangs on a pillow! And Gordon doesn’t dig with a shovel. He digs with a toothbrush!’ Once the array of possible referents was displayed, children were asked ‘What does Gordon bang on?’ Only three children failed to identify the target object during one or both of the pre-trials, and were excluded from the analysis, as noted above.

#### Sentence comprehension task

The sentence processing task was completed next. Participants sat in a dimly lit room, ~60 cm from a 1920 x 1200 LCD display. Eye movements were recorded binocularly using a Tobii X120 eye-tracker. A nine-point calibration procedure was conducted before the experiment began.

We counterbalanced the portion of the story that was referenced during the critical sentence, the order in which typical and atypical verb patients were mentioned, the pairing of stories with constraining and neutral verbs, and the location of the DCR on the screen (top right, top left, bottom right, or bottom left corner.) The location of other objects was randomized. Counterbalancing for the control condition was patterned on the experimental condition. Order of trial presentation was randomized. The test phase for the sentence comprehension task lasted approximately 8 minutes in the experimental condition, and 4 minutes in the control condition.

We also collected a number of individual difference measures. These are not reported here as they are not the focus of the current study.

#### Data calculations and scoring

The proportion of time that participants spent looking to each referent was calculated separately for two time-windows corresponding to different speech landmarks, namely the pre-naming window (1000 ms prior to verb onset), and the verb window (1267 ms prior to noun onset: that is, 1500 ms prior to noun onset, minus an additional 233 ms to allow for saccadic programming lag). This proportion was calculated by dividing looks to one of the referents by looks to all four referents and to blank space. Average looking time within these windows was calculated separately for trials with constraining verbs (for which the designation of discourse-congruent referent (DCR) and semantically congruent referent (SCR) was meaningful) and those with neutral verbs, based on gaze position measures assessed every 50 ms.

## Results

The number of successfully recorded trials and the percentage of missing trials are reported in Table 1. Sixteen constraining verb trials were dropped early in data collection due to a video timing error. Means and standard deviations for looking time measures are reported in Tables 2 and 3. For neutral verb trials, Tables 2 and 3 collapse looking times to both objects that were acted upon in an unusual way (discussed further below).

**Table 1:** Cell sample sizes in the verb window of the experimental and control conditions.

	7-year-olds			Adults		
	*N*	Trials captured	Trials not captured (% missing)	*N*	Trials captured	Trials not captured (% missing)
Experimental condition						
Constraining trials	31	86	38 (30.65)	33	97	35 (28.23)
Neutral trials	31	107	17 (13.17)	33	111	21 (16.94)
Control condition						
Constraining trials	32	96	32 (25)	33	95	37 (28.03)
Neutral verb trials	32	104	24 (19.35)	33	101	31 (25.00)

**Table 2:** Mean proportions of looking time to displayed items during the verb window: Constraining verb trials.

		7-year-olds				Adults		
Measure	*N*	*M (SD)*	Min	Max	*N*	*M (SD)*	Min	Max
Experimental condition (preceding story)								
Discourse-congruent	31	.258 (.151)	.029	.536	33	.338 (.243)	.000	.934
Semantically congruent	31	.362 (.168)	.000	.667	33	.281 (.222)	.000	.960
Atypical distractor	31	.082 (.108)	.000	.383	33	.140 (.120)	.000	.483
Typical distractor	31	.089 (.088)	.000	.313	33	.084 (.010)	.000	.399
Control condition (no preceding story)								
Discourse-congruent	32	.155 (.103)	.000	.419	33	.197 (.190)	.000	.942
Semantically congruent	32	.402 (.174)	.007	.750	33	.370 (.200)	.000	.669
Atypical distractor	32	.099 (.078)	.000	.250	33	.129 (.096)	.000	.286
Typical distractor	32	.169 (.101)	.000	.474	33	.096 (.090)	.000	.315

**Table 3:** Mean proportions of looking time to displayed items during the verb window: Neutral verb trials.

		7-year-olds				Adults		
Measure	*N*	*M (SD)*	Min	Max	*N*	*M (SD)*	Min	Max
Experimental condition (preceding story)								
Mentioned objects	31	.403 (.170)	.000	.665	33	.483 (.280)	.000	1.00
Unmentioned objects	31	.388 (.171)	.078	.691	33	.323 (.235)	.000	.762
Control condition (no preceding story)								
Mentioned objects	32	.388 (.123)	.081	.622	33	.369 (.220)	.000	.850
Unmentioned objects	32	.425 (.140)	.118	.646	33	.358 (.240)	.000	.916

Before the main analysis, we first screened for potential effects of presentation ordering resulting from the various counterbalancing procedures on the proportion of time that participants spent looking to the discourse-congruent referent (DCR) relative to the semantically-congruent referent (SCR), during the verb window. Of relevance is whether the ordering options might influence children differently than adults (e.g., children might be more likely to fixate a recently-mentioned character), in turn reducing sensitivity of the key measures. This screening was conducted using linear mixed-effect (LME) models with age and relevant trial-presentation variables (and their interactions) as predictors. Analyses were implemented using package lme4 1.1–21 in R. The ordering variables reflected: (i) the sequence in which the discourse-congruent and semantically congruent referents were mentioned (DCR first or second); (ii) the portion of the story (first or second half) to which the critical sentence referred; (iii) the verb type (constraining or neutral) of the very first trial heard by the participant; and (iv) the difference in gaze behaviour between the first two versus the final two constraining-verb trials. The dependent measure was a difference score created by subtracting the probability of fixating the DCR from the probability of fixating the SCR (probabilities were logit transformed). We used a random effects structure that was maximal in the inclusion of relevant intercept and slope terms except in testing the effect of verb type on the first trial, for which a maximal model would not converge (and some slope terms could not be included). None of the models revealed a reliable effect or interaction involving any of the trial-presentation variables (all *p*’s > .10). Thus any effects of age in the main analyses are unlikely to arise from subtle effects of stimulus ordering.

### Relative differences in the use of discourse context and semantic knowledge

We first explored relative differences in adults’ and children’s ability to use discourse context to alter the processing of a constraining verb. For this we used an LME model containing age (children: -1, adults: 1), condition (control: -1, experimental: 1) and Age x Condition as fixed effects. A maximal random effects structure was used based on participants and items as random factors, with all relevant intercept and slope terms. Separate models were conducted using the proportion of overall fixations to the SCR and overall fixations to the DCR within the window in which a constraining verb was heard.

When fixations to the SCR were the dependent measure, the results showed a marginal effect of age (*B* = -.36, *p* = .069), corresponding to adults’ overall lower likelihood of considering the SCR compared to children. There was also a significant effect of condition, with relatively less consideration of the SCR in the experimental condition (*B* = -0.6463, *p* < .001) in which the test sentences were preceded by background stories. Importantly, these effects were qualified by a significant interaction of age and condition (*B* = -0.4566, *p* = .023). Follow-up LME models conducted separately for adults and children contained condition (control: -1, experimental: 1) as a fixed effect. These models confirmed that the nature of the interaction effect is that adults reliably reduced their likelihood of fixating the SCR in the experimental condition relative to the control condition (*B* = -1.09, *p* < .001), whereas children did not (*B* = -0.19, *p* = .5).

Analyses involving looks to the DCR yielded only a main effect of condition (*B* = .811, *p* < .001), whereby child and adult listeners were less likely to look at the DCR in the control condition.

Given that the effects of the manipulations were primarily reflected in listeners’ tendency to fixate the SCR, we further conducted an exploratory analysis examining details of the timing of participants’ consideration of DCR and SCR separately after the constraining verb was encountered. Recall that all four objects mentioned during the story were displayed during the critical sentence, and participants may happen to be fixating any one of these objects at the onset of the verb window. When they are looking at a referent other than the DCR or SCR, the time that elapses before their gaze shifts to either DCR or SCR can inform us about the speed with which they first consider a semantically-driven or discourse-driven interpretation. We calculated these measures and analysed them in two LME models. First, when participants happened to be looking at a referent other than the DCR at the beginning of the verb window yet shifted to this object upon hearing the constraining verb, we found no difference in the proportion of the window that elapsed before children (coded as -1) or adults (coded as 1) shifted their gaze from that referent to the DCR (*B* = -31.24, *p* = .347; see Table 4).

**Table 4:** Mean time (ms) to shift gaze to the discourse-congruent and semantically congruent referents during the verb window in the experimental condition, constraining verb trials.

	7-year-olds (*n* = 30)			Adults (*n* = 30)		
Measure	*M (SD)*	Min	Max	*M (SD)*	Min	Max
Discourse-congruent	921 (272)	350	1267	898 (302)	150	1267
Semantically congruent	845 (238)	492	1267	977 (265)	261	1267

When participants were fixating a referent other than the SCR at the beginning of the verb window, the instances in which they shifted their gaze to the SCR showed a pattern whereby adults made slower shifts than children (*B* = 74.30, *p* = .028). Thus, not only do adults show a smaller tendency to consider the SCR compared to children, but when they do consider the SCR the accompanying gaze shift is slower than that of children. This pattern is consistent with the idea that adults are experiencing competition from the DCR even when their overt gaze is directed to the SCR.

In order to examine the use of fantastical discourse context within each age group, we now turn to an exploration of the quantitative patterns in more absolute terms. Here we use one-sample t*-*tests conducted in SPSS 21 using 1000 case resamples with replacement from the original dataset and a 95% bias-corrected and accelerated (BCa) confidence interval. This bootstrapping procedure is an appropriate method in situations where observed values violate normality or are unknown [40, 41], and allows the nonparametric calculation of a confidence interval of the estimated probability that a participant is fixating a given scene element. Nonparametric bootstrapping addresses the problem of non-normal distribution and non-homogeneity of variance and avoids the assumption of additive, as opposed to multiplicative effects [40]. Bootstrapping is not uncommon in analysis of eye-tracking data, for instance, in visual decision tasks (e.g., [42]) and in visual world studies where normality is not met (e.g., [43]), as was the case in the present study.

One-sample analyses were conducted using Holm-Bonferroni-adjusted alphas to account for the number of tests performed. This method is more powerful than the single-step Bonferroni [44]. Holm-Bonferroni corrections are performed on rank-ordered *p*-values, such that the adjusted alpha is lower for those analyses with higher *p-*values. We applied this method by grouping and ranking tests separately within age groups (children, adults) and trial types (constraining, neutral) on each dependent variable (DCR and SCR) and for each analysis window (pre-verb, verb).

### Constraining verb trials

**Fig 2:**
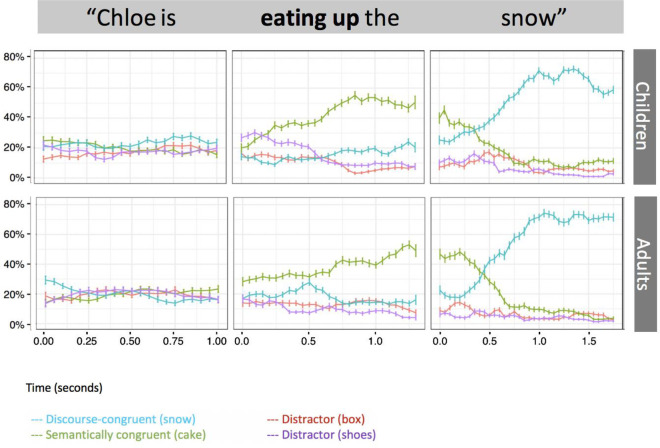
Time-course plot of proportion of looks to potential referents in the control condition on constraining verb trials. Error bars represent +/-1 standard error of the mean.

In the control condition with no preceding stories, we examined looking behaviour during the verb window on trials containing constraining verbs to identify whether listeners anticipated an object representing the most typical patient (e.g., ’cake’). Chance was calculated at .2 per referent (to account for 5 possible gaze locations: one for each referent, and additionally, blank space on the screen). One-sample t-tests demonstrated that as expected, both children and adults looked at chance (adults, *M* = .20, *SD* = .19, *p* = .926, α = .05) or at a rate below chance (children, *M* = .16, *SD* = .10, *t*(27) = -2.28, *p* = .030, α = .05, *d* = -0.43) to the DCR, and at a rate above chance to the SCR (children: *M* = .40, *SD* = .17, *t*(27) = 6.17, *p* = .001, α = .05, *d* = 1.16; adults: *M* = .37, *SD* = .20, *t*(29) = 4.67, *p* = .002, α = .05, *d =* 0.85 (Fig 2).

**Fig 3:**
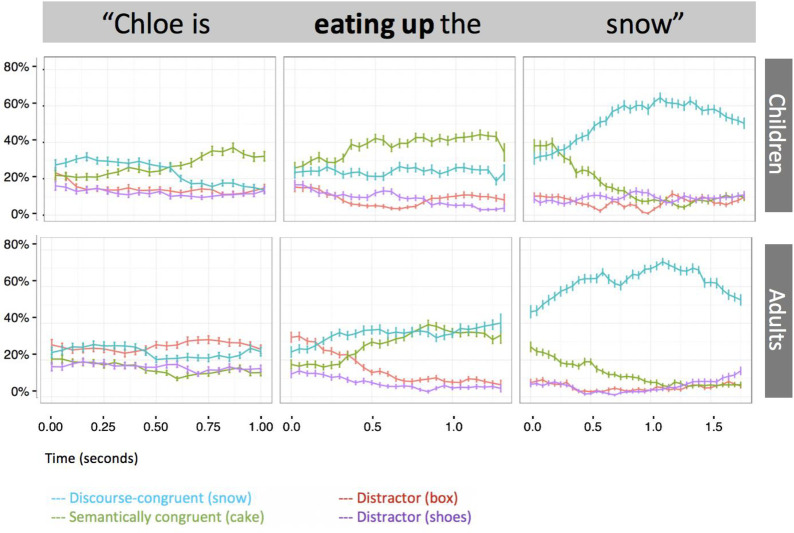
Time-course plot of proportion of looks to potential referents in the experimental condition on constraining verb trials. Error bars represent +/-1 standard error of the mean.

We next explored the pattern of effects in the experimental condition, in which sentences containing constraining verbs were preceded by fantastical stories. Recall that the displays contained two distractor objects in addition to the DCR and SCR. As a manipulation check we first ascertained whether participants looked preferentially to the DCR and SCR when treated as a class (potential patient referents), as represented by a composite measure combining gaze to both these objects. Chance was again calculated at .2 per individual referent (to account for 5 possible gaze locations: one for each referent, and additionally, blank space on the screen) and was thus set at .4 for this analysis involving the DCR and SCR together. Both adults and children looked to the DCR and SCR composite at a rate significantly above chance (children: *t*(30) = 7.07, *p* = .001, α = .016, *d* = 1.27; adults: *t*(32) = 7.83, *p* = .001, α = .016, *d* = 1.36). Thus, both children and adults discounted distractors on the basis of verb information, as seen in Fig 3.

We then turned to our more specific question, namely precisely which type of patient was anticipated by children and by adults upon hearing a constraining verb. To investigate this, we examined children’s and adults’ rates of looking against chance to establish the extent to which stored knowledge versus the fantastical discourse context influenced participants’ interpretations verb information. During the verb window, children looked to the SCR at a rate above chance (*t*(30) = 5.37, *p* = .001, α = .05, *d* = 0.964), whereas adults did not (*p* = .064, α = .05). Conversely, adults, but not children (using the corrected alpha) looked to the DCR at a rate above chance (children: *t*(30) = 2.13, *p* = .045, α = 0.025, *d* = 0.38; adults: *t*(32) = 3.27, *p* = .003, α = 0.05, *d* = 0.57). Thus, although both age groups used verb information to anticipate a particular type of “relevant” referent, only adults clearly anticipated the DCR as the patient of the constraining verb, whereas children instead anticipated the SCR.

### Neutral verb trials

**Fig 4:**
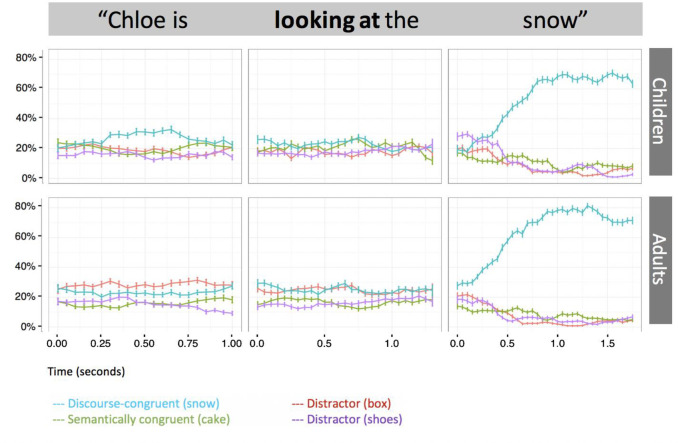
Time-course plot of proportion of looks to potential referents in the experimental condition on neutral verb trials. Error bars represent +/-1 standard error of the mean.

Recall that neutral verb trials did not contain semantically incongruent objects because the verb ("look at") was by definition compatible with all display objects. They instead contained two discourse-focused objects, in the sense that the description phase had mentioned the character carrying out two unusual actions (e.g., a fairy eating snow and putting boxes on her feet). For neutral verb trials, we therefore collapsed the proportion of looks to both of these previously-mentioned objects. As predicted, one-sample t-tests demonstrated that neither children (*M =* .40, *SD* = .17, p = .927, α = .05) nor adults (*M* = .48, *SD* = .28, *p* = .114, α = .05) made anticipatory looks to the previously-mentioned objects during the verb window of the critical sentence at a rate above chance (Fig 4). This confirmed that patterns found in the constraining verb conditions were not simply due to attentional capture or interest in the images used as the SCR and DCR. However, collapsing across constraining and neutral trials in the pre-verb window, we found that adults, but not children (*M* = .21, *SD* = .08, *p* = .408, α = .05), looked at a rate above chance to the previously mentioned objects (*M* = .25, *SD* = .11, *t*(32) = 2.499, *p* = .020, α = .05, *d* = 0.43), suggesting that adults anticipated an unusual action consistent with the story information prior to hearing the verb. Adults’ gaze toward the two mentioned objects on neutral verb trials correlated negatively with gaze toward SCR on constraining verb trials (*r*(31) = −.404, *p* = .020), suggesting that adult listeners who were relatively more able to override their semantic knowledge were those who were also more inclined to anticipate a story-relevant action in the absence of a constraining verb.

## Discussion

In the current study, we asked to what extent 7-year-old children and adults rely on stored real-world knowledge, instead of using fantastical story information, to anticipate upcoming linguistic input in real time. In the absence of a background story describing improbable events, an unfolding verb (e.g., ’eat’) led 7-year-olds as well as adults to generate expectations for an object representing a typical patient, (e.g., ’cake’, cf. [4]). However, given a fantastical story context, such as a protagonist (a fairy) who is said to have snow for her snack, expectations for the typical patient were reduced in adults, but not in children. Adults’ anticipation of the discourse-appropriate referent increased upon hearing the verb, whereas children’s did not. Adults were less likely to fixate verb patients consistent with their prior semantic and world knowledge after hearing fantastical discourse than was the case when the one-sentence discourse preceded the critical sentence, whereas children were equally likely to fixate such semantically congruent verb patients regardless of whether they had previously heard fantastical discourse information.

Adults also showed some detectable but comparatively late consideration of the semantically congruent referent, reflected in their increased anticipation of the semantically congruent referent upon hearing the verb (see the latter half of the verb window in Fig 3). This is not unexpected, however, as prediction is often accompanied by a certain degree of lexically-driven activation even when such an effect is in principle incongruent with broader sentence or discourse information [45]. Thus, predictions derived from semantic memory and from the newly-learned fantastical context were somewhat in competition even in adults. (Note that it is therefore unlikely that adults are simply better than children at deducing the purpose of the experiment.) However, when adults did generate a fixation to the semantically congruent referent, they were reliably slower than children, suggesting that for adults, discourse-coherent information competes more effectively with semantic knowledge than is the case for children. Overall, then, this study demonstrates clear age-related differences in the use of fantastical information sources to predict upcoming linguistic input.

Recall that Yazbec et al. [21] report similar age differences. Below the age of 10, children tested in their study did not privilege unexpected semantic information over their real-world knowledge during real-time sentence interpretation. Many of these sentences presented actions that were thematically plausible, though less likely than alternatives. If the under-10s tested by Yazbec et al. [21] and the 7-year-olds described here tended to rely upon their real world knowledge due to lack of strength, clarity, or maintenance of their representation of the discourse information, in turn causing it to compete poorly with stored knowledge, we might have expected a highly salient fantastical discourse to strengthen the representation and thus overcome this difficulty. A possible outcome of our study was thus that the sheer implausibility and incongruity of fantastical fictional events would bolster children’s mental representation of the discourse by drawing their attention to the contextual constraints that it imposed. In turn children would successfully use those constraints during online linguistic processing to a greater extent than has been demonstrated by studies using unexpected but often thematically plausible events [21]. We did not find this to be the case.

Our data are more consistent with the possibility that 7-year-olds’ canonical semantic and conceptual relations are so strongly rooted in the statistical patterns in language that have consolidated over time, and in the relevant real-world knowledge of typical situations of actions with which these patterns are consistent, that they tend to overwhelm new and unexpected information, even when the latter is fantastical and highly salient. This is broadly consistent with the notion that predictive language processing may be a function of two interacting systems: an automatic route, similar to Kahneman’s [46] System 1, which may draw primarily on associations and stable semantic memory; and a more effortful ‘active prediction’ route (System 2) capable of building up higher-order meaning (for further discussion in the context of a review of the nature of prediction in language processing, see [47]). On this account, an immature capacity to generate System 2 predictions could mean that 7-year-olds’ stored semantic and real-world knowledge will always be privileged relative to situation-specific information during sentence processing, irrespective of the clarity with which children represent such situation-specific information. Certain theories of linguistic prediction assume that language production implicitly supports prediction during language comprehension, and propose an association route with similarities to System 1 alongside a separate production route with similarities to System 2 (e.g., [48, 49]). More skilled producers appear to engage in more prediction (e.g., [4]); given that adults have more experience of producing language across a range of situations, they may engage in more fluent prediction-by-production and more successfully incorporate new knowledge into making predictions.

Further, a comparison of adults’ and children’s performance on neutral verb trials suggests that 7-year-olds’ responses to incongruity is not adult-like. Prior to verb onset, adults, but not children, looked at a rate above chance to the two referents involved in unusual actions stated in the earlier description phase when the verb was neutral (‘looking at’). Thus, even prior to hearing the verb, adults, but not children, anticipate an action that is relevant to the information they just heard. Our data lend preliminary support to the proposal that information that is incongruous with world knowledge signals to adults, but not to 7-year-olds, that events involving this knowledge are likely to arise shortly. While a priming account in which recent story events were more active for adults than for children cannot be fully ruled out [45], adults’ looking to the two discourse-congruent referents on neutral verb trials was negatively associated with their looking to the semantically congruent referent on constraining verb trials, suggesting that adults who more effectively overrode stored semantic knowledge had stronger expectations for a story-relevant action when no verbal cues to such an action was provided. Nonetheless, children clearly recognized the incongruity at play in our stories: despite doing a mostly excellent job on our instruction to sit still, some of our 7-year-old participants succumbed to muted giggles while listening. However, while children are enthusiastic connoisseurs of incongruity, they may not recognize it as a cue to expect further unusual events to occur later in the story. More specifically, children may not expect such further unusual events to be related to the initial incongruous event. This may reduce children’s maintenance of fantastical information in active mental representations.

Future research could address the issue of whether using longer, more repetitious, or more elaborated stories would strengthen children’s mental representations further and thus assist children in discounting stored semantic knowledge. The simplicity and brevity of our stories held children’s attention and avoided taxing their working memory, as evidenced by their success on our pre-test for children’s memory for the first half of a story. However, as demonstrated by work in developmental pragmatics [50], scaffolding children’s understanding may support their performance. More exposure to discourse information in context tends to strengthen the discourse model over time (e.g., [16]) and it is possible that at a certain duration of linguistic input and level of elaboration, the discourse model might overcome even strongly rooted canonical semantic and conceptual relations. It is also possible that 7-year-olds’ performance would be more adult-like if the new semantic relations introduced by the discourse were to be overtly legitimized. For instance, giving reasons for a character’s odd behaviour (e.g., of a snow-consuming fairy, ‘…because she can shovel it right in!’) may motivate children to construct explanations [51], mitigating inconsistency with children’s prior knowledge by linking the new semantic relation with a real-world contingency.

We investigated children’s real-time processing of fictional discourse by examining children’s ability to use a discourse context to predict upcoming referents and to override a semantically congruent interpretation. Work on children’s offline narrative comprehension has tended to focus on areas in which children lack competence compared to adult performance, rather than on explaining *how* children create meaning-based representations from narratives [52]. As a result, the development of children’s implicit processing of situation models remains poorly understood: for instance, we do not know why, although children are able to mentally represent at least some aspects of a character’s perspective during narrative comprehension by age 3 [34, 35], middle school children fail to monitor time, space, characters, goals, and causation during reading experiences [53]. A careful examination of the *real-time* processing of fantastical discourse provides a rich opportunity to characterize the information processing skills underlying children’s language comprehension at the discourse level and their mental representations of the events they hear about in narrative. It also expands our understanding of the flexibility of children’s semantic networks: by asking how children process language that strongly contradicts their knowledge of the world, and explicating the cognitive and linguistic processes involved, we can move towards a more comprehensive account of how other linguistic events that contradict children’s learned knowledge of the real world are processed in real time. Without insights into moment-by-moment changes in children’s mental representations of the events they hear about, we will be unable to achieve a comprehensive understanding of the mechanisms by which children represent and make sense of fiction, and more broadly, of counterintuitive states-of-affairs.

## Supporting information

S1 Appendix Agents, actions and objects used in each trial. Outline of the agent, action and objects used in each trial.
(DOCX)
