# Supplementary material for: Children’s and adults’ use of fictional discourse and semantic knowledge for prediction in language processing
Source: PLoS One. 2022 Apr 28;17(4):e0267297. doi: 10.1371/journal.pone.0267297 (PMC9049568; doi:10.1371/journal.pone.0267297)
Supplement: S1 Appendix — Outline of the agent, action and objects used in each trial. (DOCX) [file pone.0267297.s001.docx]

**S1 Appendix: Agents, actions and objects used in each trial**

Outline of the agent, action and objects used in each trial. In each story, the agent performed both actions, but the story concluded with a critical sentence involving only one action from the story. The portion of the story that was referenced during the critical sentence was counterbalanced. Full stories are given below the table.

| Agent | Actions | Discourse-congruent referent | Semantically congruent referent |
| --- | --- | --- | --- |
| Fairy | Eating | Snow | Cake |
|  | Putting | Boxes | Shoes |
| Elf | Riding | Vacuum | Bicycle |
|  | Putting | Pot | Hat |
| Dragon | Drinking | Paint | Juice |
|  | Sleeping | Table | Bed |
| Giant | Drawing | Ketchup | Crayons |
|  | Washing | Money | Dishes |
| Witch | Eating | Key | Sandwich |
|  | Washing | Radio | Shirts |
| Superhero | Drinking | Soap | Milk |
|  | Drawing | Blanket | Paper |
| Monster | Riding | Garbage can | Truck |
|  | Climbing | Shelves | Ladder |
| Robot | Sleeping | Bathtub | Couch |
|  | Climbing | Lamp | Stairs |

Chloe the fairy doesn’t have cake for her snack.

She has snow for her snack!

And Chloe doesn’t wear shoes on her feet.

She wears boxes on her feet!

What is Chloe going to do?

Chloe is eating up the snow / Chloe is putting on the box.

Elliott the elf doesn’t get around on a bicycle.

He gets around on a vacuum!

And Elliott doesn’t wear hats.

He wears pots!

What will Elliott do next?

Elliott is riding on the vacuum / Elliott is putting on the pot.

Danny the dragon likes to have a glass of paint!

He doesn’t like juice.

And at night, Danny doesn't lie down on a bed.

He lies down on a table!

What is Danny going to do?

Danny is drinking up the paint / Danny is sleeping on the table.

Tom the giant doesn't use crayons for colouring.

He uses ketchup for colouring!

And Tom doesn't put his dishes in the sink.

He puts his money in the sink!

What will Tom do next?

Tom is drawing with the ketchup / Tom is washing up the money.

Rachel the robot never takes naps on the couch.

She takes naps in the bathtub!

And Rachel goes up lamps!

She doesn't go up stairs.

What is Rachel going to do?

Rachel is sleeping in the bathtub / Rachel is climbing up the stairs.

Charlie the superhero likes to have a sip of soap!

He doesn't like to have milk.

And Charlie paints on blankets!

He doesn't paint on paper.

What will Charlie do next?

Charlie is drinking up the soap / Charlie is drawing on the blanket.

Fred the monster doesn't drive a truck.

He drives a garbage can!

And Fred walks up shelves!

He doesn't walk up ladders.

What is Fred going to do?

Fred is riding in the garbage can / Fred is climbing up the shelves.

Wendy the witch has keys for her lunch!

She doesn't have sandwiches for her lunch.

And Wendy puts her radio in the laundry!

She doesn't put her shirts in the laundry.

What will Wendy do next?

Wendy is eating up the key / Wendy is washing up the radio.
